# The short-term outcomes of pulmonary metastasectomy or stereotactic body radiation therapy for pulmonary metastasis from epithelial tumors

**DOI:** 10.1186/s13019-020-1079-4

**Published:** 2020-02-27

**Authors:** Ryu Kanzaki, Osamu Suzuki, Takashi Kanou, Naoko Ose, Soichiro Funaki, Yasushi Shintani, Masato Minami, Keisuke Tamari, Keisuke Otani, Yuji Seo, Fumiaki Isohashi, Kazuhiko Ogawa, Meinoshin Okumura

**Affiliations:** 1 Department of General Thoracic Surgery, Osaka University Graduate School of Medicine, L5-2-2 Yamadaoka, Suita-city, Osaka, 565-0871 Japan; 2 Department of Radiation Therapy, Osaka University Graduate School of Medicine, Suita, Japan; 3 Department of General Thoracic Surgery, National Hospital Organization Toneyama Hospital, Toyonaka, Japan

**Keywords:** Pulmonary metastasis, Surgery, Stereotactic body radiation therapy

## Abstract

**Background:**

Stereotactic body radiation therapy (SBRT) has recently been widely performed for relatively small-volume tumors. We analyzed the short-term outcomes of pulmonary metastasectomy (PM) or SBRT for pulmonary metastases.

**Methods:**

This study was a retrospective analysis of 82 patients with pulmonary metastasis from epithelial tumors who underwent PM or SBRT between 2013 and 2016.

**Results:**

Fifty-nine patients underwent PM, 21 patients underwent SBRT, and 2 patients underwent combined PM and SBRT. The mean age of the PM group was significantly lower than that of the SBRT group (60.6 vs 67.4 years, *p* = 0.03). The most frequent types of primary tumor in the PM and SBRT groups were colorectal cancer (*n* = 27, 46%) and head and neck squamous cell carcinoma (*n* = 8, 38%), respectively. The rate of treatment-associated complications did not differ between the two groups to a statistically significant extent (20% vs 24%, *p* = 0.76). The 3-year local control rates of the two groups were similar (PM group, 88%; SBRT group, 92%; *p* = 0.48). The 3-year progression-free survival (PFS) rate of the PM group were better than that of SBRT groups (42% vs 11%, *p* = 0.01). The 3-year overall survival (OS) rates of the PM and SBRT groups were 77 and 52% respectively; although the rate of the PM group was higher, the difference was not statistically significant (*p* = 0.10).

**Conclusions:**

SBRT provides a favorable 3-year local control rate. The 3-year OS rate of the SBRT group tended to be lower than that of the PM group, despite the difference was not statistically significant. PM and SBRT play complementary roles in patients with pulmonary metastases.

## Background

Surgical resection of pulmonary metastases from various types of cancers is currently the first choice of treatment for patients who can tolerate surgery [1]. However, the evidence to support the efficacy of pulmonary metastasectomy (PM) is weak; there have been no prospective randomized studies to compare PM with other treatment options [2].

Recently, SBRT has been widely performed for small sized primary lung cancer or pulmonary metastasis [3–6]. In stage IA primary lung cancer patients who are considered unfit for surgery, the cancer-specific survival outcomes of SBRT and surgery are reported to be comparable. For pulmonary metastases, some reports have demonstrated a favorable short-term local control rate [7, 8]. At present, SBRT is performed for the treatment of primary lung cancer and pulmonary metastases for cases in which surgery is deemed intolerable [3–6].

Despite the evolving role of SBRT in the treatment of pulmonary metastases, few reports have compared the outcomes of PM and SBRT at a single institution. In addition, the treatment of metastatic lesions is changing due to recent advances in molecular targeted therapy for various types of cancer [9]. Thus, there is a need for studies utilizing the latest data on the treatment of pulmonary metastases. In this background, we analyzed the short-term outcomes of PM or SBRT for pulmonary metastases in the current era.

### Patients and methods

The present study was a retrospective analysis of 82 consecutive patients who underwent PM or SBRT in our hospital between 2013 and 2016. The study protocol was approved by the Ethical Review Board for Clinical Studies at Osaka University (control number 17259). Patients with pulmonary metastasis from lung cancer were excluded from this study because it is often difficult to distinguish second primary lung cancer from pulmonary metastasis from primary lung cancer. Patients with pulmonary metastasis from non-epithelial tumors (*n* = 17 who underwent PM and n = 1 who underwent SBRT) were also excluded. When pulmonary metastasis from various tumors was detected, the treatment strategy was decided by the cancer board of our hospital. The patients who met the following criteria underwent PM [10]: 1) the pulmonary nodule was deemed completely resectable; 2) the absence of apparent mediastinal lymph node metastasis on a preoperative radiological examination; 3) the metastatic disease was limited to the lungs or extrapulmonary distant metastasis was controlled or controllable if present; 4) locoregional control of the primary tumor was achieved; and 5) the patient was in good overall general condition and their respiratory function was sufficient to tolerate lung resection. The standard first-choice treatment mode for pulmonary metastasis was PM. SBRT was recommended for patients who had some factors contraindicating surgery, including older age, a compromised general condition, a poor respiratory function, a lung nodule in an unfavorable central location, or if the patient refused to undergo surgery. The criteria for SBRT is as follows: 1) the largest diameter of pulmonary nodule(s) was not more than 5 cm, 2) the number of pulmonary nodules is no more than three, 3) no extrapulmonary distant metastasis or controlled if present, 4) no apparent findings of interstitial lung diseases. Generally, patients were followed-up with computed tomography for at least 3 months before PM or SBRT to exclude patients with rapidly progressive disease.

When PM was performed, the type of resection was selected according to the size and location of the tumor, the overall general condition, and the respiratory function of the patient; lesser resection was preferably selected as long as curative resection was possible. In terms of the surgical approach, video assisted thoracoscopic surgery (VATS) was the first choice. Thoracotomy was selected when the preoperative computed tomography (CT) findings suggested that the palpation of the nodule during surgery was necessary for detecting the pulmonary nodule or assuring a sufficient margin. All of the specimens obtained from pulmonary resection were reviewed by pathologists.

SBRT was performed using a dedicated CyberKnife G4 system according to our institutional treatment protocol. A total dose of 52 Gy in 4 fractions was administered to cover the D99 of the gross tumor volume (GTV).

The treatment dose was adapted to the risk of toxicity: 60Gy in 10 fractions was given if the all or part of a lesion was found within the two centimeters around the central proximal airways, as defined in RTOG protocols. The averaged delivered dose D95 of planned target volume (PTV) was 43.5(+ − 2.3) Gy which was prescribed at 70 (+ − 7.4) % of the maximum dose which leads to irradiate center portion of tumor with higher dose. Treatment planning was based on 4D-CT, and accounted for respiratory movements. Patient positioning was verified based on offline CT and corrected online at each fraction using the TLS system equipped in the CyberKnife system.

Follow-up was generally based on the findings from chest CT, a physical examination, and laboratory blood tests performed every 6–12 months after treatment. Follow-up information was obtained from the hospital medical records. The time interval between pulmonary resection and the latest follow-up in the present study ranged from 1 to 67 months (median: 28 months). In the present study, local progression was defined as ipsilateral intra thoracic recurrence with the exception of another pulmonary metastasis; i.e., local progression of the pulmonary nodule after SBRT, surgical margin relapse after surgery, pleural dissemination, or lymph node metastasis. Local control rate is defined as the rate of free from local progression.

The statistical analyses were performed using the JMP Pro 13 software program (SAS Institute, Berkley, CA, USA). The data are expressed as the mean ± the standard deviation (SD). Differences in clinical variables between the two groups were evaluated using Student’s t-test or the chi-squared test. The survival rates were analyzed with the Kaplan-Meier method, using the date of pulmonary resection or start of SBRT as the starting point. *P* values of < 0.05 were considered to indicate statistical significance.

## Results

During the study period, 59 patients underwent PM (PM group), and 23 patients underwent SBRT. Of the 23 patients who underwent SBRT, 2 patients had undergone PM for the first pulmonary recurrence before the study period. After the exclusion of these 2 patients, the 21 remaining patients were classified into the SBRT group. The characteristics of the patients in the two groups are shown in Table 1. The mean age of the patients in the PM group was significantly lower than that of the patients in the SBRT group (60.6 vs 67.4 years, *p* = 0.03). The most frequent types of primary tumor in the PM and SBRT groups were colorectal cancer (CRC) (*n* = 27, 46%) and head and neck squamous cell carcinoma (HNSCC) (*n* = 8, 38%), respectively. With regard to the mode of treatment for the primary tumor, 58 patients (98%) in the PM group and 11 patients (52%) in the SBRT group underwent treatment that included surgery (*p* < 0.01). The disease-free interval (DFI), number of lesions, and the size of lesions in the two groups did not differ to a statistically significant extent.

**Table 1:** Patient characteristics

Characteristics	PM group (*n* = 59)	SBRT group (*n* = 21)	*P* value
Sex			
Male	35 (59%)	14 (67%)	
Female	24 (41%)	7 (33%)	N.S.
Age (years)			
Mean ± SD	60.6 ± 14.6	67.4 ± 10.9	0.03
Range	28–84	39–82	
Primary tumor			
Colorectal cancer	27 (46%)	6 (29%)	
Gynecologic malignancies	8 (14%)	2 (10%)	
Head and neck squamous cell carcinoma	3 (5%)	8 (38%)	
Renal cell carcinoma	7 (12%)	0 (0%)	
Salivary gland tumor	3 (5%)	0 (0%)	
Breast cancer	1 (2%)	2 (10%)	
Esophageal cancer	3 (5%)	2 (10%)	
Others	7 (12%)	1 (5%)	< 0.01
Treatment mode for primary tumor			
Treatment includes surgery	58 (98%)	11 (52%)	
Chemoradiotherapy or radiotherapy	1 (2%)	10 (48%)	< 0.01
Disease free interval (months)			
Mean	28	27	
Range	0–128	0–111	
0(synchronous)	11 (19%)	2 (10%)	
1–12	13 (22%)	6 (29%)	
13–24	7 (12%)	5 (24%)	
24-	28 (47%)	8 (38%)	N.S.
No. of lesions			
1	45 (76%)	15 (72%)	
2	10 (17%)	3 (14%)	
3	3 (5%)	3 (14%)	
4	1 (2%)	0 (0%)	N.S.
Size of target lesion (cm)			
Mean ± SD	1.6 ± 1.1	1.6 ± 0.6	N.S.
Range	0.6–8.5	0.9–3.1	
Metastasis besides lung at the time of pulmonary metastasectomy or SBRT	15 (25%)	7 (33%)	N.S.
Prior chemotherapy for metastatic disease	16 (27%)	7 (33%)	N.S.

*PM* pulmonary metastasectomy, *SBRT* stereotactic body radiation therapy, *SD* standard deviation

The details of the PM group are described in Table 2. Fifty-one patients (86%) underwent VATS, and 38 patients (64%) underwent sublobar resection. Lymph node metastasis was pathologically confirmed in 2 patients (3%).

**Table 2:** Details of pulmonary metastasectomy

Characteristics	No. of patients
Approach	
Thoracotomy	8 (14%)
VATS	51 (86%)
Type of resection	
Bilobectomy	1 (2%)
Lobectomy	20 (34%)
Segmentectomy	10 (17%)
Partial resection	28 (47%)
Lymph node dissection	
Not done	32 (54%)
Sampling	12 (20%)
Hilar	13 (22%)
Mediastinal	2 (3%)

*VATS* video assisted thoracic surgery

Treatment-associated complications occurred in 12 patients in the PM group (20%) and 5 patients in the SBRT group (24%) and did not differ to a statistically significant extent (*p* = 0.76). The treatment-associated complications that occurred in the PM group included prolonged air leak (*n* = 4), arrhythmia (*n* = 2), pneumonia (*n* = 1), an acute exacerbation of interstitial pneumonia (*n* = 1), postoperative bleeding (n = 1) and other complications (*n* = 3). The treatment-associated complications that occurred in the SBRT group were radiation-induced pneumonitis (*n* = 4) and pneumonia (n = 1). Postoperative chemotherapy were performed in 19 patients in PM group and 5 patients in SBRT group.

**Fig. 1:**
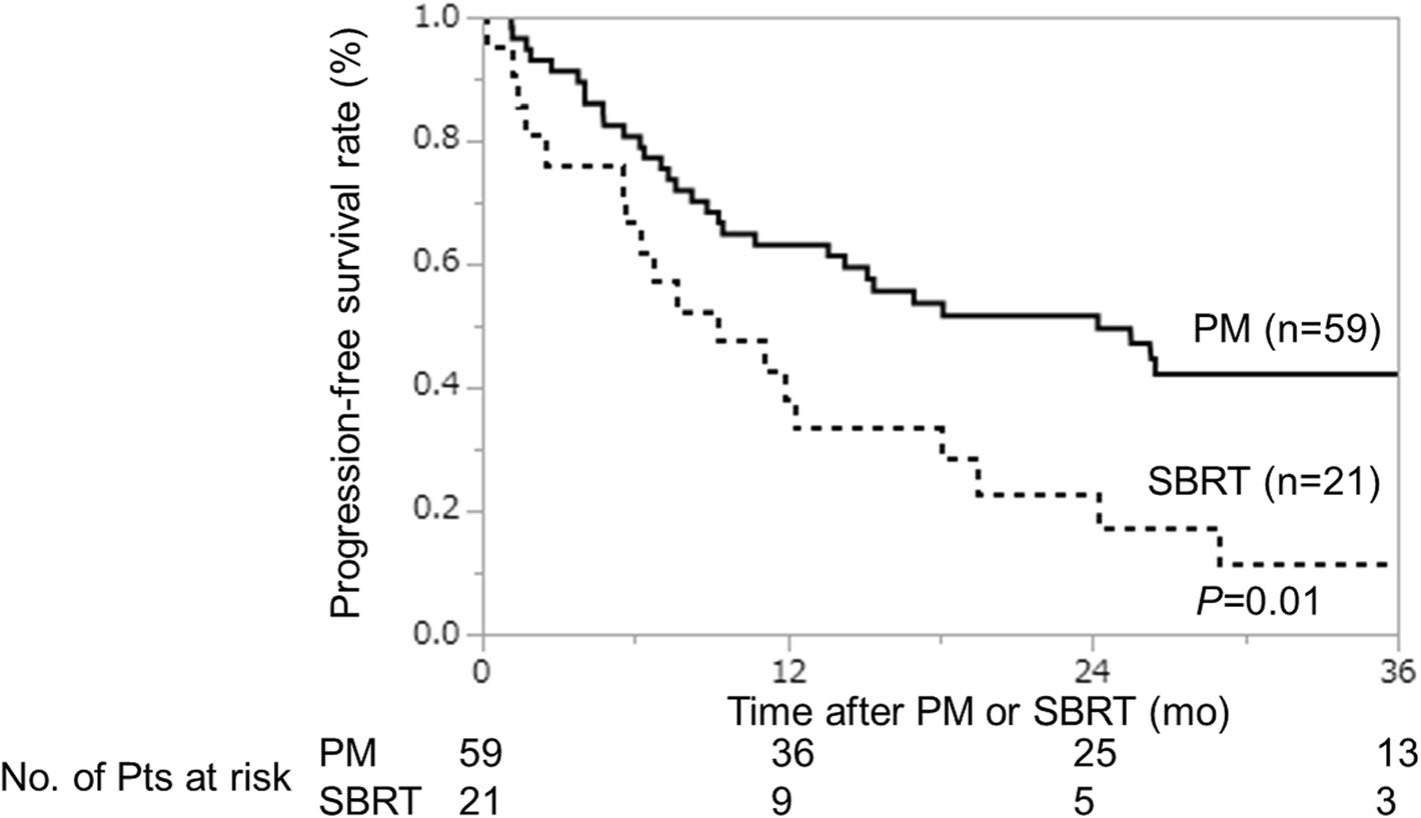
The 3-year progression free survival rates of the PM group and SBRT groups. The 3-year progression-free survival rate of the PM group were better than that of SBRT groups (42% vs 11%, *p* = 0.01)

**Fig. 2:**
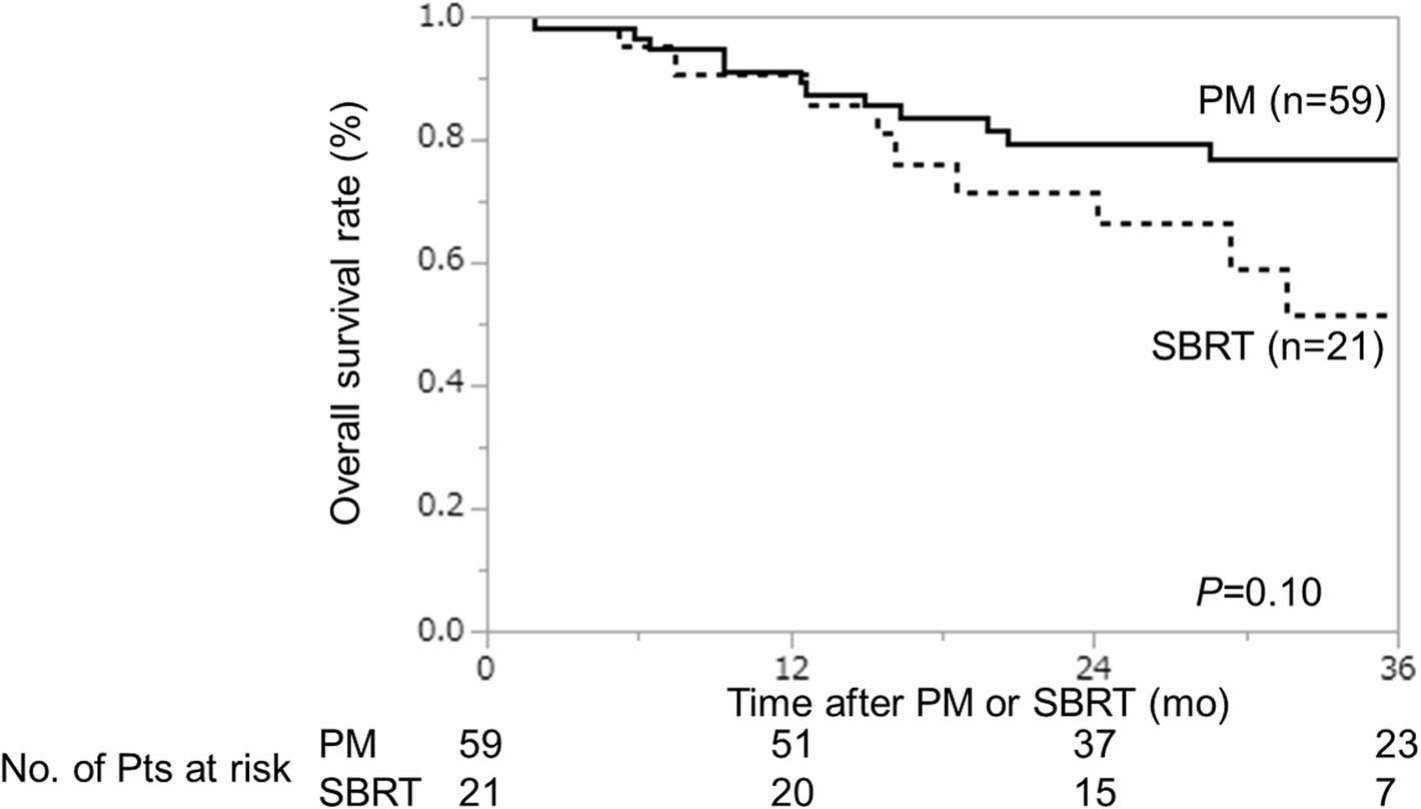
The 3-year overall survival rates of the PM and SBRT groups. The 3-year overall survival rates of the PM and SBRT groups were 77 and 52%, respectively, and did not differ to a statistically significant extent (*p* = 0.10)

Thus far, 14 patients were censored because these patients are followed by the other hospitals, 45 patients are alive and 21 patients were dead, and 32 of the 59 patients in the PM group and 18 of the 21 patients in the SBRT group have experienced recurrence. The sites of recurrence in the two groups are shown in Table 3. The most frequent site of recurrence was distant metastasis besides lung in the PM group. On the otherhand, lung alone, distant metastasis besides lung, and local relapse of the primary tumor or regional lymph nodes are almost equally distributed in the SBRT group. Five patients in the PM group experienced local progression (pleural dissemination, *n* = 4; surgical margin relapse, *n* = 1) while 1 patient experienced local progression of the pulmonary nodule after SBRT. The two groups had a similar 3-year local control rate (PM group, 88%; SBRT group, 92%; *p* = 0.48). The 3-year progression-free survival (PFS) rate of the PM group were better than that of SBRT groups (42% vs 11%, *p* = 0.01) (Fig. 1). The 3-year overall survival (OS) rates of the PM and SBRT groups were 77 and 52% respectively; although the value of the PM group was higher, the difference was not statistically significant (*p* = 0.10) (Fig. 2).

**Table 3:** Site of recurrence

	Patients experienced recurrence after PM (*n* = 32)	Patients experienced recurrence after SBRT (*n* = 18)
Lung alone	11 (34%)	6 (33%)
Distant metastasis besides lung	13 (41%)	6 (33%)
Local relapse of primary tumor or regional lymph nodes	3 (9%)	5 (28%)
Local progression of PM alone	4 (13%)	0 (0%)
Local progression of PM and distant metastasis	1 (3%)	1 (6%)

*PM* pulmonary metastasectomy, *SBRT* stereotactic body radiation therapy

The details of the 2 patients who underwent SBRT during the study period and who had been undergone PM before the study period are shown in Table 4; both patients underwent PM and SBRT, and favorable short-term outcomes were achieved.

**Table 4:** Details of the patients who underwent SBRT during the study period and had been undergone PM before the study period

Age	Sex	Primary tumor	DFI (months)	History of treatment for pulmonary metastasis	Location	Local control	Outcome
64	M	Bladder cancer	134	Conventional RT for RUL, partial resection of LLL	RUL, central	46 months yes	46 months NED
45	F	Colorectal cancer	36	Right middle lobectomy, partial resection of LLL	RLL, central	40 months yes	13 months repeat SBRT for recurrent pulmonary metastasis, 40 months NED

*DFI* disease free interval, *LLL* left lower lobe, *NED* no evidence of disease, *PM* pulmonary metastasectomy, *RLL* right lower lobe, *RT* radiotherapy, *RUL* right upper lobe, *SBRT* stereotactic body radiation therapy

## Discussion

In the present study, we analyzed the short-term outcomes of PM or SBRT for the treatment of pulmonary metastases. The rate of treatment-associated complications did not differ to a statistically significant extent, and the 3-year local control rates of the two groups were similar. The 3-year OS rate of the SBRT group tended to be lower than that of the PM group, despite the difference was not statistically significant.

With regard to the comparison of SBRT and PM for pulmonary metastases, Lodeweges et al. recently reported the long-term outcomes [11]. In their report, the 5-year OS rates of PM and SBRT were 41 and 45%, respectively, and the 5-year local control rates were 81 and 83% respectively. The 3-year OS rate of the SBRT group tended to be lower than that of the PM group, despite the difference was not statistically significant. Future study with large number of patients is needed to evaluate the survival difference of these two groups.

Because SBRT is currently indicated for patients who are deemed medically unfit for PM, a direct comparison of PM and SBRT is theoretically impossible. In the present study, the characteristics of the patients in the PM and SBRT groups were different (i.e., the patients in the SBRT group were older, and the types of primary tumor were different). PM is an established treatment, and less-invasive surgery is commonly performed in the current era. It has been reported that the long-term outcomes of thoracoscopic surgery are not inferior to those of open surgery [12]. To prevent relapse at the surgical margin, it is important to ensure a sufficient and cytologically-negative surgical margin [13]. In our institute, intraoperative rapid margin cytology is routinely performed when segmentectomy or partial resection is performed [14]. In the present study, we only experienced one patient with relapse at the surgical margin. We believe that the intraoperative performance of rapid margin cytology is useful for reducing the rate of relapse at the surgical margin. In terms of local control of SBRT, the local control rate of pulmonary metastasis from CRC in patients who receive SBRT is reported to be inferior to that of other types of cancer [15]. Recently, a dose escalation study achieved excellent local control in spite of metastatic CRC [16]. The optimal dosage for such patients should be determined in prospective study.

Remarkably, 4 patients (7%) in the PM group suffered from pleural dissemination; in contrast, none of the patients in the SBRT group suffered from pleural dissemination. Based on these data, it is possible that surgery itself causes pleural dissemination. This is an important issue to be solved in the future. The rate of treatment-associated complications did not differ to a statistically significant extent. In the SBRT group, the most notable complication was radiation-induced pneumonitis. It is reported that chronic obstructive pulmonary disease (COPD) and the Brinkman index are predictors of prolonged minimal radiation-induced pneumonitis [17]. Radiation oncologists should take care to detect the development of radiation-induced pneumonitis when treating such patients.

Because the patient characteristics differs according to the type of primary cancer, the treatment strategy for pulmonary metastases should be discussed for each tumor type [1]. In the SBRT group, the most frequent type of cancer was HNSCC, followed by CRC. The general condition of the HNSCC patients tended to be poor. In our experience, a low body mass index (< 18.5), a history of malignancy besides HNSCC, and COPD were each found to be significantly associated with the development of postoperative complications when pulmonary resection was performed for patients with a history of HNSCC [18]. Thus, it is considered that SBRT may be a good alternative choice of treatment for these patients. Based on our experience, postoperative complications, such as prolonged air leak, were found to occur relatively frequently when lobectomy was performed for patients with a history of esophageal cancer [19]. Then, it is thought that patients with centrally located pulmonary metastasis from esophageal cancer would be good candidates for SBRT as well. Future study is needed to provide evidence of treatment options for these patients.

A proportion of patients with pulmonary metastasis develop recurrent pulmonary metastasis after local treatment and repeat pulmonary resection is effective for these patients [20, 21]. However, the 2nd or 3rd pulmonary resection is difficult to be performed for patients with a poor general condition, a decreased respiratory function, or a centrally located tumor that requires lobectomy. We believe that SBRT is beneficial for these patients. As demonstrated in Table 4, we experienced cases in which both PM and SBRT were successfully performed. Both treatment modalities played complementary roles in these patients.

At any rate, the most important issue in the treatment of metastatic cancer is whether a local treatment strategy or a systemic treatment strategy is superior. In the current era, molecular-targeted personalized therapy is evolving [9, 22]. In these circumstances, the role of local treatment for metastatic disease should be continuously verified.

The present study is associated with some limitations. The study population was relatively small and the follow up period was relatively short. Further studies should be performed to investigate the long-term outcomes. Because the indication criteria for PM and SBRT are different, information based on direct comparison of these two groups should be carefully interpreted.

## Conclusion

SBRT provided a favorable 3-year local control rate. The 3-year OS rate of the SBRT group tended to be lower than that of the PM group, despite the difference was not statistically significant. PM and SBRT can play complementary roles in patients with pulmonary metastases.

## Data Availability

All the data used in the present study are preserved in Department of General Thoracic Surgery, Osaka University Graduate School of Medicine and are available from the corresponding author on reasonable request.

## Abbreviations

COPD: Chronic obstructive pulmonary disease
CRC: Colorectal cancer
CT: Computed tomography
DFI: Disease-free interval
GTV: Gross tumor volume
HNSCC: Head and neck squamous cell carcinoma
LLL: Left lower lobe
N.S.: Not specific
NED: No evidence of disease
OS: Overall survival
PFS: Progression-free survival
PM: Pulmonary metastasectomy
PTV: Planned target volume
RLL: Right lower lobe
RT: Radiotherapy
RUL: Right upper lobe
SBRT: Stereotactic body radiation therapy
SD: Standard deviation
